# A novel *STK11* gene mutation (c.388dupG, p.Glu130Glyfs∗33) in a Peutz-Jeghers family and evidence of higher gastric cancer susceptibility associated with alterations in STK11 region aa 107-170

**DOI:** 10.1016/j.gendis.2021.11.002

**Published:** 2021-11-19

**Authors:** Giovanna Forte, Filomena Cariola, Katia De Marco, Andrea Manghisi, Filomena Anna Guglielmi, Raffaele Armentano, Giuseppe Lippolis, Pietro Giorgio, Cristiano Simone, Vittoria Disciglio

**Affiliations:** a Medical Genetics, National Institute of Gastroenterology “S. de Bellis” Research Hospital, Castellana Grotte, Bari 70013, Italy; b Histopathology Unit, National Institute of Gastroenterology “S. de Bellis,” Research Hospital, Castellana Grotte, Bari 70013, Italy; c Department of General Surgery, National Institute of Gastroenterology “S. de Bellis” Research Hospital, Castellana Grotte, Bari 70013, Italy; d Gastroenterology and Digestive Endoscopy Unit, National Institute of Gastroenterology “S. de Bellis” Research Hospital, Castellana Grotte, Bari 70013, Italy; e Department of Biomedical Sciences and Human Oncology (DIMO), Medical Genetics, University of Bari Aldo Moro, Piazza Umberto I, Bari 70124, Italy

Peutz-Jeghers syndrome (PJS) is a rare autosomal dominant disorder characterized by mucocutaneous pigmentation and gastrointestinal (GI) hamartomatous polyposis and is associated with an increased risk of gastrointestinal, breast, gynecologic and other extra-GI malignancies. The serine/threonine kinase 11 (*STK11*) gene has been identified as a pathogenic factor in PJS. *STK11* is a tumor suppressor gene located on chromosome 19p13.3 and includes 9 coding exons.^1^ The STK11 protein is composed of 433 amino acids (aa) and comprises a kinase catalytic region (aa 49–309) as well as N- and C-terminal regulatory domains. The catalytic region is further divided into 3 functional domains: an ATP-binding and orientation domain (aa 49–106), the catalytic site (aa 123–148), and a substrate-binding domain (aa 171–225).^2^ STK11 plays an important role in tumorigenesis since it is implicated in numerous key biological processes involving cell metabolism, cell cycle regulation, cell polarity and motility, and angiogenesis. *STK11* germline mutations have a wide genetic heterogeneity, and genotype–phenotype associations are not yet clearly established.^2^ Here we report a novel frameshift mutation involving the *STK11* gene (c.388dupG, p.Glu130Glyfs∗33) in an Italian family, in which this mutation is associated with gastric lesions. In light of this finding, we performed a meta-analysis to ascertain whether *STK11* nonsense and frameshift germline mutations may affect gastric cancer (GC) susceptibility in PJS patients based on their location. These data revealed a trend toward a higher risk of developing GC in PJS patients with *STK11* truncating mutations in region aa 107–170.

In the current study, we examined an Italian family with a clinical diagnosis of PJS. The index case (Fig. 1A II:28) is a 42-year-old woman with several GI hamartomatous polyps, which was referred to our institute for genetic counseling. The patient underwent esophagogastroduodenoscopy (EGD) due to persistent vomiting events at 42 years of age, which revealed erythematous gastritis in the stomach. Histological examination of gastric mucosa biopsies showed moderate chronic atrophic gastritis in the antrum and body of the stomach caused by *Helicobacter pylori*. Moreover, a pedunculated polyp (25 mm) and many sessile polyps (4–5 mm) were detected by EGD in the second portion of the duodenum. A follow-up colonoscopy performed one month later identified numerous pedunculated and sessile polyps in the colon (3–40 mm), seven of which were surgically removed. Histological examination of a polyp surgically excised from the left colic flexure revealed a sessile tubular adenoma with low-grade dysplasia. Moreover, the other polyps were histologically classified as likely hamartomatous lesions. Subsequently, a computed tomography (CT) scan showed many pedunculated polyps in the gastric antrum, duodenal bulb, jejunal loops, ileum, and proximal ascending colon. The patient had a positive family history of cancer and gastrointestinal polyposis. Her father died at 64 years of age from gastric cancer (Fig. 1A I:12). A paternal uncle developed colorectal cancer at 65 years of age (Fig. 1A I:4). Moreover, a paternal aunt and two other paternal uncles (Fig. 1A I:3; I:5; I:6) developed intestinal polyps between 52 and 58 years of age. The index patient underwent a molecular study of the *STK11* gene (Supplementary Methods). This analysis identified a novel heterozygous frameshift variant, c.388dupG, in exon 3 (Fig. 1B). This variant was not detected in the healthy first-degree relatives tested (Fig. 1A II:27; II:29). As indicated above, the father (Fig. 1A I:12) and four paternal relatives (one aunt and three uncles) (Fig. 1A I:3, I:4, I:5, I:6) of the index patient presented with the classic phenotypic spectrum of PJS, but they were not willing to undergo *STK11* molecular analysis. *STK11* c.388dupG was found to be rare since it was not listed in global population databases. Moreover, this variant has never been reported in major disease-associated databases (HGMD Professional and ClinVar). More than 400 *STK11* germline mutations are reported in the literature, most of which are frameshift or nonsense alterations resulting in an abnormal truncated protein and the loss of kinase activity. Missense mutations, small insertions or deletions, and whole gene deletions have also been reported.^1^^,^^2^ The *STK11* c.388dupG mutation is located in the core of the catalytic site (aa 123–148) and causes a frameshift in the coding sequence at codon 130, leading to premature termination of translation at codon 163 (p.Glu130Glyfs∗33), with loss of the STK11 catalytic domain and C-terminal non-catalytic regulatory region. The loss of STK11 kinase activity has been reported as a major cause of cancer susceptibility in PJS.^1^ Indeed, one of the main roles of STK11 is to activate AMPK via phosphorylation of Thr172. Under metabolic stress, active AMPK causes p53-dependent G1 cell cycle arrest and reduces protein synthesis and cell growth through the mTOR pathway.^1^ Therefore, a possible explanation for the pathogenic effect of the c.388dupG mutation is that the loss of STK11 catalytic domain and the consequent impairment of its kinase activity may affect AMPK activation, thereby contributing to polyp formation and tumorigenesis.

**Figure 1:**
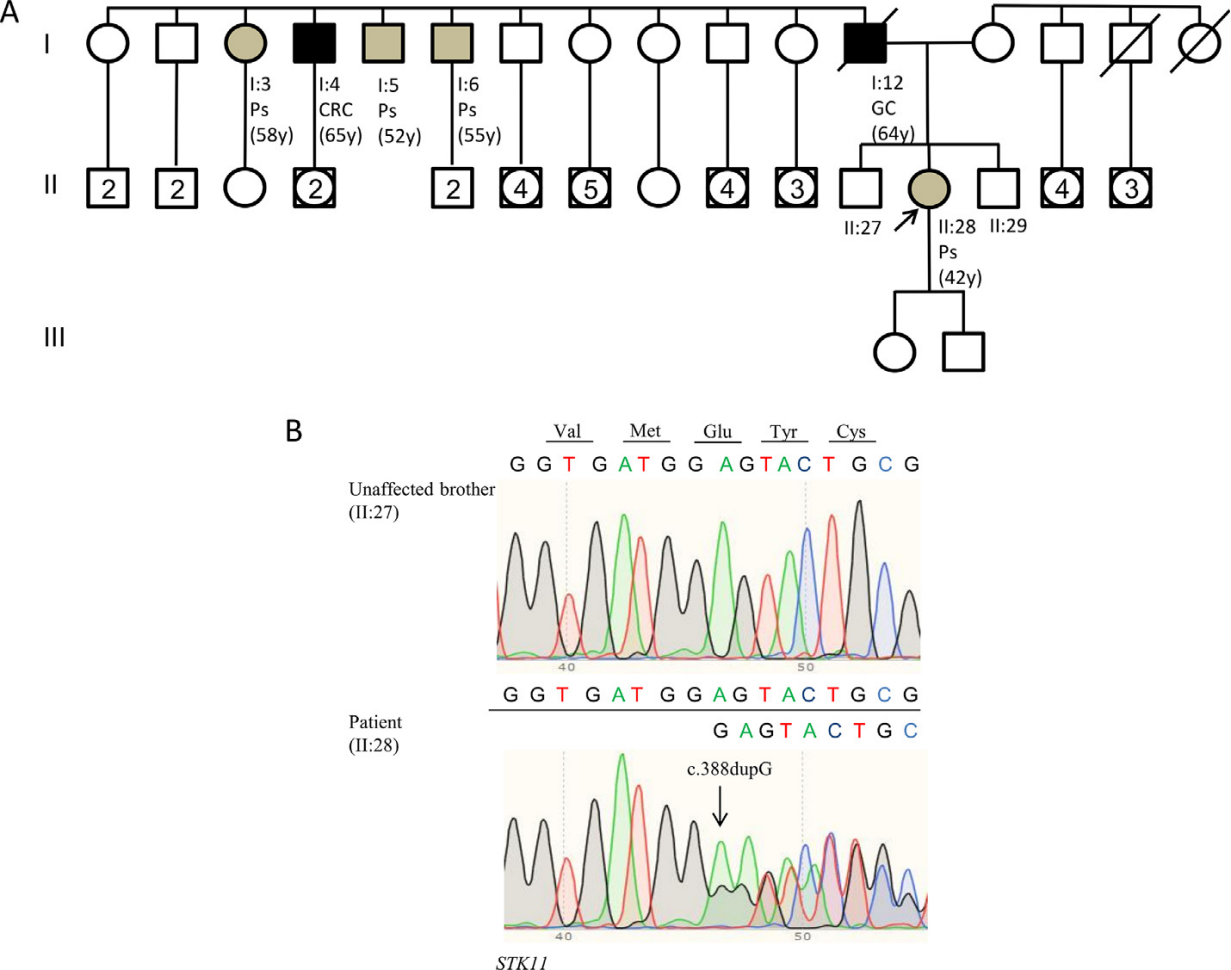
Genetic characteristics of gene mutation. **(A)** Pedigree of the family involved in this study. Squares indicate men, circles represent women. Squares and circles with a number inside represent multiple individuals. The arrow indicates the index case. Unfilled symbols indicate unaffected individuals. Slashed symbols denote dead individuals. Black-filled symbols denote individuals with cancer, while grey-filled symbols correspond to patients with gastrointestinal polyps. The following information is given below each filled symbol: clinical manifestations (CRC = colorectal cancer; GC = gastric cancer; Ps = gastrointestinal polyps), age at diagnosis (y = years). **(B)** Sequencing electropherograms of genomic DNA from the index patient and an unaffected brother, showing the c.388dupG (p.Glu130Glyfs∗33) mutation.

The HGMD Professional database only reports 23 mutations in STK11 catalytic site (aa 123–148), 19 of which result in a truncated protein. Among these 19 mutations, 11 were associated with clinical information. Only one of these mutations has been characterized at the functional level in a paper by Chen et al.^3^ Specifically, the authors analyzed an *STK11* frameshift mutation that leads to premature protein truncation at codon 162 (p.Arg147Leufs∗15), showing that it causes decreased wild-type STK11 protein levels in circulating leucocytes compared to unaffected relatives, which suggests STK11 haploinsufficiency. This reduction in STK11 protein levels was found to be higher in PJS polyps when compared with normal tissue, indicating that epigenetic regulation of STK11 expression may contribute to polyp formation. Moreover, the authors showed that reduced STK11 protein expression was correlated with decreased AMPK Thr172 phosphorylation levels.^3^ Since the *STK11* c.388dupG mutation identified in our study leads to premature protein truncation at codon 163 (p.Glu130Glyfs∗33), *i.e.*, one amino acid downstream compared to the mutation reported by Chen and colleagues, it is tempting to speculate that, besides decreasing AMPK Thr172 phosphorylation levels, it may also cause STK11 haploinsufficiency. Furthermore, STK11 subcellular localization is essential for its tumor suppressor activity through the regulation of growth suppression. STK11 shuttles from the nucleus to the cytoplasm by interacting with STRADα/β-MO25α/β complexes. The whole STK11 kinase catalytic domain, as well as aa 319–343, are essential for STRADα/β binding, while MO25α/β is a scaffold that stabilizes this interaction.^3^ Of note, eleven STRADα isoforms are expressed in the GI tract, where PJS clinical manifestations primarily occur. Of these, only STRADα-iso3 can bind to and activate STK11, inducing its cytoplasmic retention, and is thus a key regulator of STK11-mediated intestinal epithelial cell polarization.^4^ In addition, several mutant forms of STK11 identified in PJS patients localize only in the nucleus and are not detectable in the cytoplasm, emphasizing the importance of STK11 cytoplasmic localization.^5^ Therefore, it can be hypothesized that the p.Glu130Glyfs∗33 STK11 truncated protein, which lacks the C-terminal region, is unable to bind to STRADα/β-MO25α/β complexes and to be anchored into the cytoplasm. Altogether, this evidence suggests that *STK11* c.388dupG may be involved in the impaired regulation of key cellular processes, ultimately leading to the formation of PJS polyps.

Based on the family history of gastric lesions reported in the examined Italian family, we sought to correlate the location of *STK11* nonsense and frameshift mutations with gastric cancer susceptibility by performing a meta-analysis of published studies comprising relevant clinical data. This analysis showed that 83 unique *STK11* truncating germline mutations located in the kinase catalytic region (aa 49–309), including the one characterized in this study, were identified in 137 PJS patients (Fig. S1A). Within this subset of 83 truncating mutations, 14 were located in the ATP-binding and orientation domain (aa 49–106), 21 were located in the catalytic site and its flanking regions (aa 107–170), and 48 were located in the C-terminal portion of the catalytic domain (aa 171–309) (Fig. S1A). Among these 83 truncating mutations, 27 have been associated with gastric lesions in a total of 30/137 patients (Table S1). This analysis did not reveal a statistically significant association between the gastric polyp phenotype and the location of *STK11* mutations. Indeed, we found that gastric polyps were detected in 3 out of 26 patients (11.5%) harboring a truncating mutation in STK11 region aa 49–106, in 4 out of 41 patients (9.7%) harboring a truncating mutation in STK11 region aa 107–170, and in 13 out of 70 patients (18.6%) harboring a truncating mutation in STK11 region aa 171–309. Importantly, this analysis revealed that the percentage of PJS patients with GC harboring *STK11* mutations in region aa 107–170 was significantly higher (7/41, 17%) than the percentage of patients with *STK11* mutations in region aa 49–106 (0/26, 0%) (*P* = 0.0375) or region aa 171–309 (3/70, 4.2%) (*P* = 0.0437) (Fig. S1B). Thus, the results of our meta-analysis showed a trend toward GC susceptibility in patients with nonsense and frameshift mutations in STK11 region aa 107–170 compared with patients harboring mutations in the remaining kinase catalytic region.

In this study, we identified a novel heterozygous *STK11* frameshift mutation (c.388dupG) causing premature termination of translation at codon 163 (p.Glu130Glyfs∗33), which contributes to the PJS phenotype. This novel mutation in the *STK11* gene expands the mutational spectrum of PJS. Furthermore, we found that PJS patients carrying *STK11* nonsense and frameshift germline mutations in region aa 107–170 seem to have increased susceptibility to develop GC. Further studies characterizing the mutational status and clinical manifestations of a larger number of PJS patients are necessary to define the exact correlation between GC and the location of *STK11* mutations. This finding may have a significant impact on the surveillance, tailored management, and overall quality of life of PJS patients.

## Conflict of interests

The authors declare no conflict of interest.

## Funding

This work was supported by the 10.13039/501100005010Italian Association for Cancer Research (IG grant N. 23794 to C.S.), by the Italian Ministry of Health ‘‘Ricerca Corrente 2018–2020; 2019–2021’’ to C.S., and by the 10.13039/501100003407Italian Ministry of Education, University and Research (MIUR) ‘‘PRIN - Research Projects of National Relevance’’ (PRIN 2017, N. 2017WNKSLRLS4) to C.S.

## Appendix A Supplementary data

Multimedia component 2 Multimedia component 2

Multimedia component 3 Mutation distribution throughout *STK1*. **(A)** Distribution of nonsense and frameshift mutations throughout *STK11* kinase catalytic region (aa 49–409) identified in our meta-analysis of PJS patients. Triangles represent mutations. Filled symbols indicate mutations associated with gastric cancer (black) and gastric polyps (grey). **(B)** Distributions of PJS cases (percentage) with gastric cancer and gastric polyps based on the location of *STK11* mutations in the different functional domains. Multimedia component 3
