# Supplementary material for: A novel STK11 gene mutation (c.388dupG, p.Glu130Glyfs∗33) in a Peutz-Jeghers family and evidence of higher gastric cancer susceptibility associated with alterations in STK11 region aa 107-170
Source: Genes Dis. 2021 Nov 19;9(2):288–91. doi: 10.1016/j.gendis.2021.11.002 (PMC8843985; doi:10.1016/j.gendis.2021.11.002)
Supplement: Multimedia component 1 [file mmc1.docx]

# Supplementary Methods

Patient recruitment

Informed consent for genetic analysis was obtained from all subjects analyzed, using a form approved by the competent ethics committee. This study was conducted in full accordance with the principles of the Declaration of Helsinki and any other applicable local ethical and legal requirements (protocol code n. 170, date of approval 31 October 2016). Molecular testing carried out in this study is based on the routine clinical diagnostic assessment performed at our Institute.

Genetic analysis

The STK11 gene was analyzed by Sanger sequencing. Briefly, genomic DNA was extracted from peripheral blood with the QIAamp DNA Blood Mini Kit (Qiagen, Carlsbad, CA, USA) according to the manufacturer’s instructions. *STK11* complete coding region (9 exons) was screened for mutations. The sequences of primers used for PCR and sequencing are available upon request. Each amplicon was sequenced in forward and reverse directions with the same primers used for PCR amplification. PCR sequencing and capillary electrophoresis were performed on an Applied Biosystems 3130 Genetic Analyzer (Thermo Fisher Scientific, Waltham, MA, USA). Mutations and polymorphisms were confirmed in independently amplified PCR products. The global population frequency of the identified STK11 variant was retrieved from the 1000 Genome (<https://www.ncbi.nlm.nih.gov/variation/tools/1000genomes/>), dbSNP (<https://www.ncbi.nlm.nih.gov/snp/?cmd=search>), gnomAD (<https://gnomad.broadinstitute.org/>), and NHLBI Exome Sequencing Project (ESP) (<https://evs.gs.washington.edu/EVS/>) databases. Moreover, the HGMD Professional (<http://www.hgmd.cf.ac.uk/ac/index.php>) and ClinVar (<https://www.ncbi.nlm.nih.gov/clinvar/>) databases were interrogated to assess the pathogenicity of the identified variant. The variant was classified according to the American College of Medical Genetics and Genomics (ACMG) and Association of Molecular Pathology (AMP) variant classification scheme.

Meta-analysis

The meta-analysis was performed on the Human Gene Mutation Database Professional (HGMD Professional), a comprehensive collection of germline mutations in nuclear genes that are associated with human-inherited diseases. We focused on the *STK11* kinase catalytic region (aa 49-409) to identify an association between truncating *STK11* mutations and gastric cancer. We reviewed all the papers identified in the aforementioned database and collected clinical information (i.e., age, sex, gastric polyps, and gastric cancer) concerning patients with nonsense and frameshift mutations in the *STK11* gene. Mutations without clinical information were excluded. Fisher exact test was performed using the free statistical software R. Fisher exact test was used to compare the clinical features (gastric lesions) in patients with truncating mutations involving the STK11 regions***:*** aa 49-106, which comprise the ATP-binding and orientation domain; aa 107-170, which include the catalytic site; and aa 171-309, which contain the substrate-binding domain).
