# Supplementary material for: A novel STK11 gene mutation (c.388dupG, p.Glu130Glyfs∗33) in a Peutz-Jeghers family and evidence of higher gastric cancer susceptibility associated with alterations in STK11 region aa 107-170
Source: Genes Dis. 2021 Nov 19;9(2):288–91. doi: 10.1016/j.gendis.2021.11.002 (PMC8843985; doi:10.1016/j.gendis.2021.11.002)
Supplement: Multimedia component 2 [file mmc2.docx]

| Supplementary Table 1. Clinical and molecular data of PJS patients with gastric lesions. | | | | | | | |  |  |
| --- | --- | --- | --- | --- | --- | --- | --- | --- | --- |
| Index patient | | | | | | | **Affected relatives** | | Ref. |
| PJS case | **Exon** | **Mutation (nucleotide)** | **Effect** | **Domain** | **Age and sex** | **Gastric lesions** | **PJS cases in family**  **(incl. index patient)** | **Gastric lesions in relatives** |  |
| HP04 6213 | 1 | 180C>A | Y60X | ATP-BINDING AND ORIENTATION | 22, M | P | 1 | - | 1 |
| 3 | 1 | 180delC | Y60X | ATP-BINDING AND ORIENTATION | 3, M | P | 1 | - | 2 |
| Patient 1 | 1 | 182delG | G61AfsX63 | ATP-BINDING AND ORIENTATION | 14, M | P | 1 | - | 3 |
| 1216 | 2 | 350insTTTG | 117fs |  | - | P | 2 | - | 4 |
| 7F | 2 | 351dupA | Y118IfsX45 |  | - | - | 2 | C | 5 |
| 419 | 2 | 354C>A | Y118X |  | - | P | 1 | - | 4 |
| II:28 | 3 | 388dupG | E130GfsX33 | CATALYTIC SITE | 42, F | P | 2 | C | This paper |
| - | 3 | 418delC | - | CATALYTIC SITE | - | C | 1 | - | 6 |
| PJ56 | 3 | 426_428del | V143fsX144 | CATALYTIC SITE | 40, F | - | 2 | C | 7 |
| PJS 056 | 3 | 426delC | S142Rfs | CATALYTIC SITE | 8, F | - | 1 | C | 8 |
| II-7 | 3 | 440_441delGT | R147LfsX15 | CATALYTIC SITE | 38, M | P | 5 | - | 9 |
| - | 3 | 465insG | - |  | - | C | 1 | - | 6 |
| 65/1/1 | 4 | 508C>T | Q170* |  | 18, F | - | 6 | C | 10 |
| HP09 6853 | 4 | 540delC | N181TfsX107 | SUBSTRATE BINDING | 35, M | P | 1 | - | 1 |
| HP07 6525 | 4 | 550delC | L184SfsX103 | SUBSTRATE BINDING | 10, M | P | 1 | - | 1 |
| F2 III-5 | 5 | 717G>A | W239* |  | 37, F | P | 2 | P | 11 |
| PJ-9 | 6 | 748dupA | - |  | 18, F | P | 1 |  | 12 |
| 626 | 6 | 789del4 | 263FS |  | - | P | 1 | - | 4 |
| PJ47 | 6 | 815-816insA | Y272X |  | 31, F | P | 1 | - | 7 |
| 2295 | 6 | 841del | 281FS |  | - | P | 4 | - | 4 |
| PJ51 | 6 | 842-844del | L282fsX5 |  | 38, M | P | 1 | - | 7 |
| PJ-1 | 6 | 843insG | - |  | 11, M | P | 1 | - | 12 |
| F3 III-1 | 7 | 871G>T | E291* |  | 4, M | P | 4 | - | 11 |
| HP05 6456 | 7 | 876C>G | Y292X |  | 22, F | - | 2 | P | 1 |
| II-3 | 7 | 890delG | R297fsX38 |  | 49, M | C | 6 | C | 13 |
| 23F | 7 | 892_893insC | F298SfsX20 |  | - | - | 2 | C | 5 |
| PJ-7 | 8 | 923G>A | W308X |  | 11, F | P | 1 | - | 12 |

P, polyps; C, cancer

**Supplementary References**

1. Papp J, Kovacs ME, Solyom S, Kasler M, Børresen-Dale AL, Olah E. High prevalence of germline STK11 mutations in Hungarian Peutz-Jeghers Syndrome patients. BMC Med Genet 2010 Nov;11:169.
2. Chow E, Meldrum CJ, Crooks R, Macrae F, Spigelman AD, Scott RJ. An updated mutation spectrum in an Australian series of PJS patients provides further evidence for only one gene locus. Clin Genet 2006 Nov;70:409-14.
3. Ausavarat S, Leoyklang P, Vejchapipat P, Chongsrisawat V, Suphapeetiporn K, Shotelersuk V. Novel mutations in the STK11 gene in Thai patients with Peutz-Jeghers syndrome. World J Gastroenterol 2009 Nov;15: 5364–5367.
4. Olschwang S, Boisson C, Thomas G. Peutz-Jeghers families unlinked to STK11/LKB1 gene mutations are highly predisposed to primitive biliary adenocarcinoma. J Med Genet 2001 Jun;38:356-60.
5. Wang Z, Wu B, Mosig, Yulan Chen RA, Ye F, Zhang Y, Gong W, Gong L, Huang F, Wang X, Nie B, Zheng H, Cui M, Wang Y, Wang J, Chen C, Polydorides AD, Zhang DY, Martignetti JA, Jiang B. STK11 domain XI mutations: candidate genetic drivers leading to the development of dysplastic polyps in Peutz-Jeghers syndrome. Hum Mutat 2014 Jul;35:851-8.
6. Mehenni H, Resta N, Park JG, Miyaki M, Guanti G, Costanza MC. Cancer risks in LKB1 germline mutation carriers. Gut 2006;55:984–990.
7. Lim W, Hearle N, Shah B, Murday V, Hodgson SV, Lucassen A, Eccles D, Talbot I, Neale K, Lim AG, O'Donohue J, Donaldson A, Macdonald RC, Young ID, Robinson MH, Lee PWR, Stoodley BJ, Tomlinson I, Alderson D, Holbrook AG, Vyas S, Swarbrick ET, Lewis AAM, Phillips RKS, Houlston RS. Further observations on LKB1/STK11 status and cancer risk in Peutz–Jeghers syndrome. British Journal of Cancer 2003 Jul;89:308–313.
8. Hearle N, Schumacher V, Menko FH, Olschwang S, Boardman LA, Gille JJP, Keller JJ, Westerman AM, Scott RJ, Lim W, Trimbath JD, Giardiello FM, Gruber SB, Offerhaus GJA, Rooij FWMDE, Wilson JHP, Hansmann A, Möslein G, Royer-Pokora B, Vogel T, Phillips RKS, Spigelman AD, Houlston RS. STK11 status and intussusception risk in Peutz-Jeghers syndrome. J Med Genet 2006 Aug;43:e41.
9. Chen JH, Zheng JJ, Guo Q, Liu C, Luo B, Tang SB, Cheng JD, Huang EW. A novel mutation in the STK11 gene causes heritable Peutz-Jeghers syndrome - a case report. BMC Medical Genetics 2017 Feb;18.
10. Schumacher V, Vogel T, Leube B, Driemel C, Goecke T, Möslein G, Royer-Pokora1 B. STK11 genotyping and cancer risk in Peutz-Jeghers syndrome. J Med Genet 2005 May;42:428-35.
11. Fu J, Wen Z, Wang F, Zhong W, He Q, Liang Q, Zhang S, Kuang Y, Liu X, Zhu D, Yu J, Qiu X, Xia H. Genetic and Clinical Analyses of Southern Chinese Children with Peutz-Jeghers Syndrome. Genet Test Mol Biomarkers 2015 Sep;19:528-31.
12. Chiang JM, Chenbc TC. Clinical manifestations and STK11 germline mutations in Taiwanese patients with Peutz–Jeghers syndrome. Asian Journal of Surgery 2018, Sep, 41: 480-485.
13. Shinmara K, Goto M, Tao MH, Shimizu S, Otsuki Y, Kobayashi H, Ushida S, Suzuki K, Tsuneyoshi T, Sugimura H. A novel STK11 germline mutation in two siblings with Peutz-Jeghers syndrome complicated by primary gastric cancer. Clin Genet 2005 Jan;67:81-6.
