# Supplementary figures and images for: A novel STK11 gene mutation (c.388dupG, p.Glu130Glyfs∗33) in a Peutz-Jeghers family and evidence of higher gastric cancer susceptibility associated with alterations in STK11 region aa 107-170
Source: Genes Dis. 2021 Nov 19;9(2):288–91. doi: 10.1016/j.gendis.2021.11.002 (PMC8843985; doi:10.1016/j.gendis.2021.11.002)

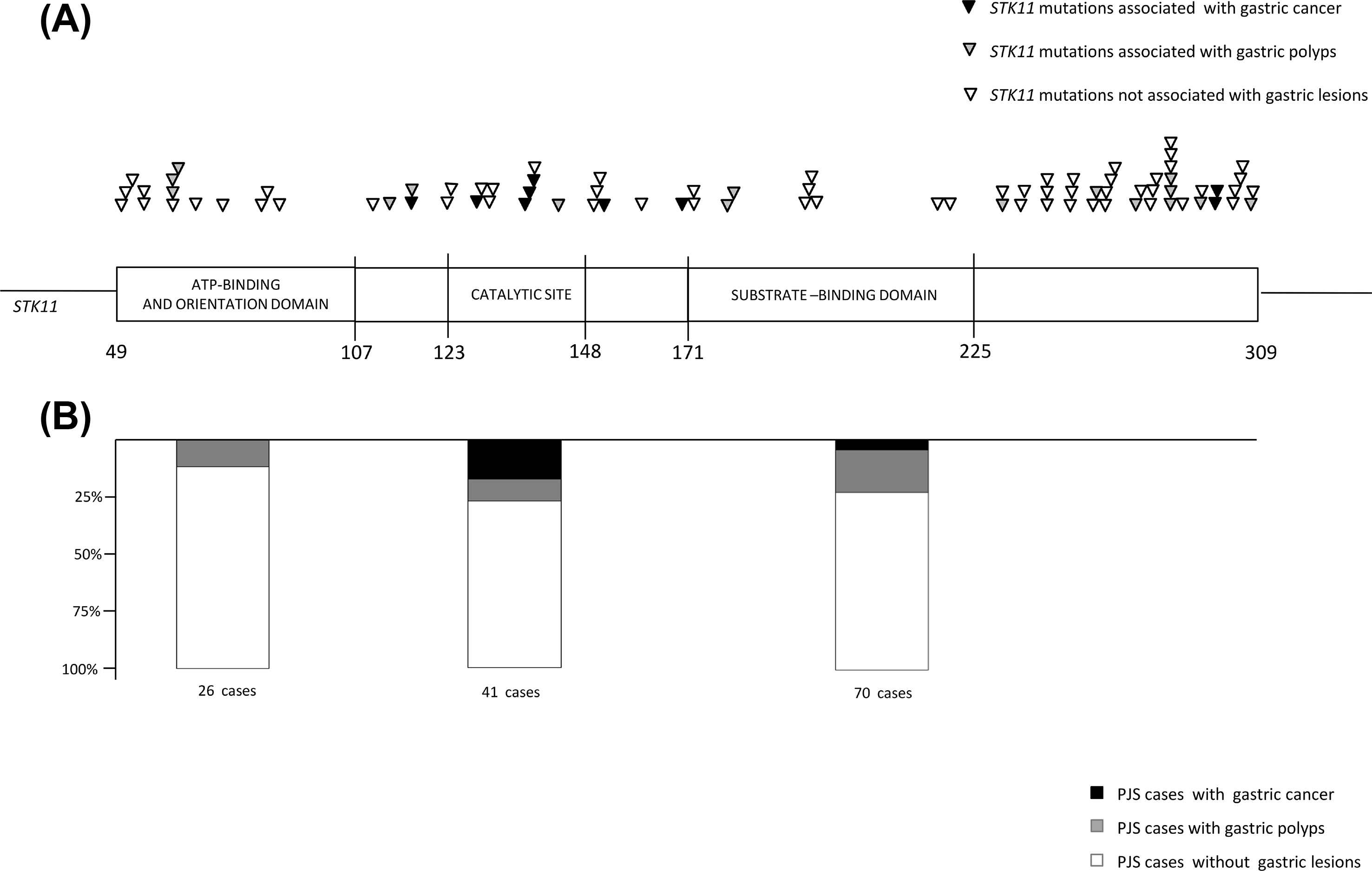

Supplement: Multimedia component 3 — Mutation distribution throughout STK1. (A) Distribution of nonsense and frameshift mutations throughout STK11 kinase catalytic region (aa 49–409) identified in our meta-analysis of PJS patients. Triangles represent mutations. Filled symbols indicate mutations associated with gastric cancer (black) and gastric polyps (grey). (B) Distributions of PJS cases (percentage) with gastric cancer and gastric polyps based on the location of STK11 mutations in the different functional domains. [file figs1.jpg]
